# Paratubal Cystectomy in a Pregnant Woman Using the Single-Incision Laparoscopic Surgery (SILS) Technique

**DOI:** 10.1155/2022/2802767

**Published:** 2022-07-14

**Authors:** Luke William Nelson, Elliot MacKenzie

**Affiliations:** Dunedin Public Hospital, 201 Great King Street, Dunedin 9010, New Zealand

## Abstract

**Introduction:**

The proliferation of prenatal ultrasound has enhanced the detection of adnexal masses during pregnancy. The presentation necessitates a clear approach to investigation and treatment that balances both maternal and fetal risk. Laparoscopy is a safe approach to surgical management in the pregnant patient, and SILS may contribute to minimising perioperative complications. *Case Presentation*. We present the case of a 21-year-old female in her second trimester of pregnancy presenting with a large 20 cm right adnexal cyst. We proceeded with laparoscopic cystectomy via the SILS technique. There were no intraoperative complications, and she recovered well postoperatively.

**Conclusion:**

Laparoscopic resection of adnexal lesions is safe during pregnancy and should be favoured over the open approach. SILS minimises incision sites and has potential for reduction in perioperative morbidity.

## 1. Introduction

The proliferation of prenatal ultrasound has enhanced the detection of adnexal masses during pregnancy. With an incidence of 2%, the presentation necessitates a clear approach to investigation and treatment that balances both maternal and fetal risk [1]. In determining the appropriate management strategy, namely, intervention vs. conservative, there are considerations idiosyncratic to the pregnant patient that should be accounted for. The absence of malignant features on imaging and stable size may obviate the need for intervention and allow for conservative management [2].

Historical concerns regarding safety of laparoscopy in pregnancy have now largely been assuaged. There still exist considerations unique to the pregnant patient. For example, the gravid uterus may be susceptible to puncture during blind insufflation [3].

Single-incision laparoscopic surgery (SILS) is an emerging approach to laparoscopy. It confers improved cosmesis and, by limiting the surgery to a single-entry point, reduces the potential for infection and bleeding [4]. Its chief disadvantages include a greater technical challenge and longer operative duration [5].

We present a case report of a 21-year-old pregnant woman who underwent a right paratubal cystectomy at 18 weeks gestation using a SILS approach.

## 2. Case Presentation

Our patient was a 21-year-old female who presented with a wanted pregnancy at 14 + 3 weeks gestation complicated by abdominal pain and vomiting. She had a medical history of 2 previous first trimester miscarriages and a raised body mass index of 33. She was taking appropriate folic acid supplementation. On examination, she had a palpable mass in the right lower abdominal quadrant, with associated tenderness and guarding. A vaginal examination was unremarkable, and the cervical os was closed. She had normal blood pressure and pulse. Her serum hCG was 15900.

**Figure 1:**
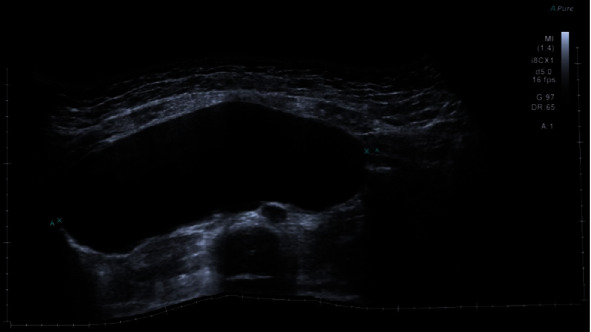
Transverse ultrasound view of the right adnexa, demonstrating the cyst. Note its homogenous echotexture.

She proceeded to have a pelvic ultrasound which demonstrated a single live intrauterine pregnancy consistent with a gestational age of 14 + 3 weeks and a large right adnexal mass measuring 201 × 148 × 73 mm (1129 cc), likely arising from the right ovary. There were no septations, solid components, or abnormal vascularity noted (Figure 1).

**Figure 2:**
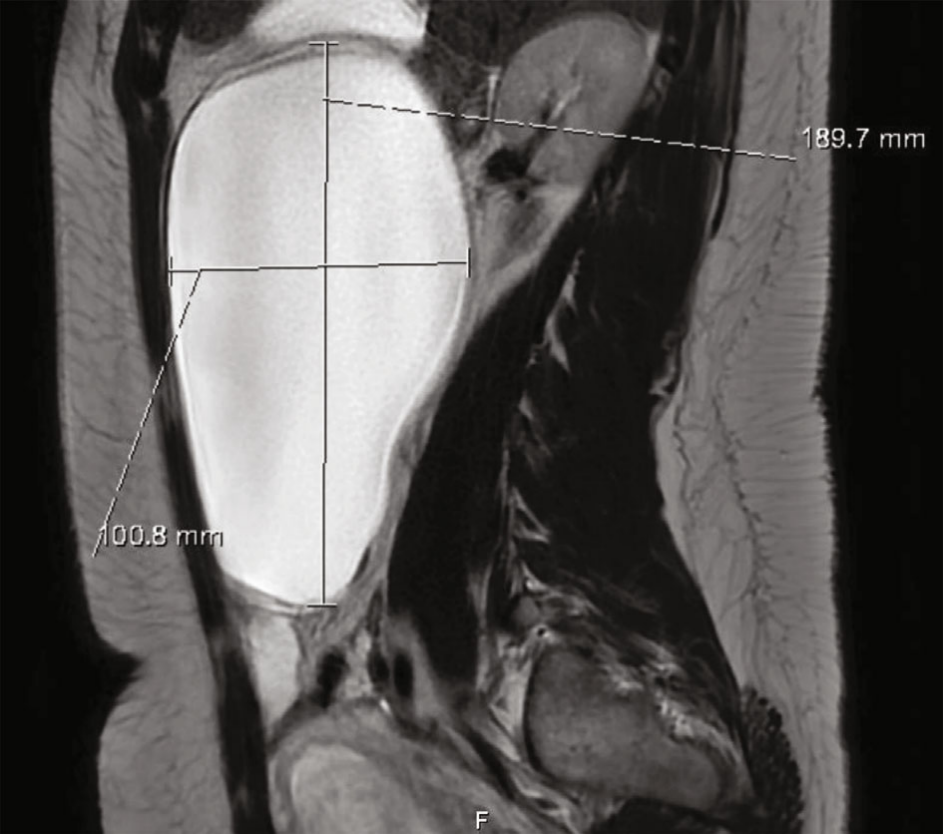
Sagittal view (MRI) of the right adnexal cyst. Note its simple appearance.

She was admitted overnight for analgesia and antiemesis and discharged the next day following improvement in her symptoms. A planned MRI was conducted as an outpatient when she was 17 + 2 weeks gestation (Figure 2). This demonstrated interval growth of the cyst from the previous ultrasound scan and concurred with the presumptive diagnosis of a large right simple cyst measuring 190 × 170 × 100 mm (1680 cc).

The patient was referred to our gynaecological-oncology multidisciplinary team. It was determined that the cyst was benign in appearance, but surgical resection was necessitated. Cystectomy was favoured over drainage, for concerns regarding reaccumulation.

**Figure 3:**
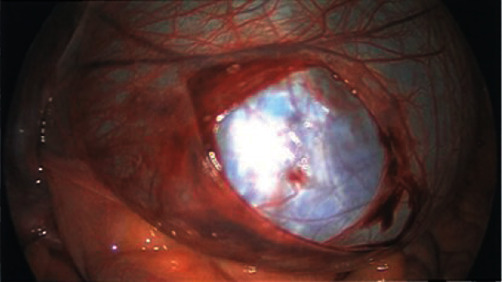
The partially dissected right adnexal cyst.

We proceeded to take her to theatre for laparoscopic right adnexal cystectomy at 18 + 2 weeks gestation. The operation was conducted under general anaesthesia by endotracheal intubation in Trendelenburg position with a urinary catheter in situ. Due to her gravid state, no transvaginal uterine manipulation was performed. Following skin preparation and application of sterile drapes, we made a 15 mm incision at the umbilicus. A SILS port (Applied Medical Gelseal) was placed at the umbilicus, through which we insufflated the peritoneal cavity with carbon dioxide. Via the SILS port, a 30-degree laparoscope and 2 working instruments were inserted. A large right adnexal mass extending to reach the liver was visualised and determined to be paratubal in origin (Figure 3).

**Figure 4:**
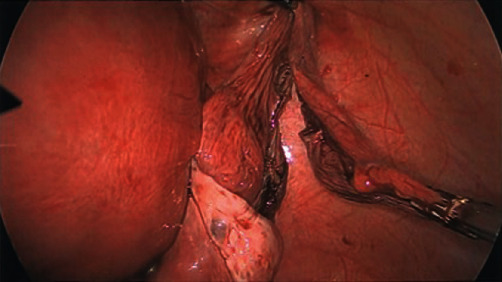
The right adnexa following complete excision of the cyst. Note the normal right ovary and gravid uterus.

Both ovaries and left fallopian tube were normal. Peritoneal washings were taken. A sharp incision was made on the cyst capsule followed by water-jet hydrodissection using a standard laparoscopic suction irrigation system under pressure. Following near complete dissection within its capsule, leaving base attached to maintain orientation, the cyst was punctured sharply with laparoscopic scissors under suction tip and suction immediately placed into cyst cavity. 1300 millilitres of clear serous fluid were drained without spill. Patient size, gravid uterus, and instrument length then limited reach deep into pelvis from the umbilicus position to allow cyst excision and control of haemostasis at its base via the SILS port alone; hence, an accessory 5 mm port was placed in the left flank. We then proceeded to complete the excision of the cyst capsule using Ligasure bipolar device, delivering it extracorporeally via the SILS port. The right-sided fallopian tube was elongated and partially twisted. We untwisted the right fallopian tube following the cystectomy. Otherwise, both the right and left fallopian tubes and ovaries were normal and left intact bilaterally (Figure 4).

Pneumoperitoneum was released, and the SILS port was removed from the abdomen. The rectus sheath at the umbilicus was sutured with 0 Vicryl, and overlying skin was sutured with 3/0 Monocryl. The left-sided accessory port site was repaired with 3/0 Monocryl.

The estimated blood loss was approximately 75 millilitres, and no intraoperative complications arose. She had an uneventful postoperative course and was discharged the next day.

Histology confirmed the diagnosis of a benign serous cystadenoma. The patient was followed up at 22 + 2 weeks gestation, and her pregnancy was progressing well.

## 3. Discussion

The finding of an ovarian cyst during pregnancy presents a dilemma to the obstetrician. Should it be necessitated, surgery must be used judiciously and timed so that fetal risk is minimised. Amongst such patients, there is a preponderance of functional cysts. This is reflected by a 70% spontaneous resolution rate by the 2^nd^ trimester [6]. Nevertheless, 1-6% of adnexal masses in pregnancy are malignant and necessitate timely resection and staging [7].

### 3.1. Diagnosis and Selection for Surgery

Ultrasound is the mainstay of investigation of ovarian masses in pregnancy, allowing the obstetrician to stratify patients according to features suggestive of malignancy [8]. Tumour markers such as the glycoprotein CA-125 are of reduced applicability in the pregnant patient and may rise as a consequence of pregnancy itself [9, 10]. MRI can provide a useful adjunct to diagnosis, through further characterisation of mass morphology [11]. Both methods are safe and do not expose the fetus to the effects of ionising radiation [12].

Our patient had radiographic features highly suggestive of a benign simple cyst. However, imaging alone cannot fully replace pathological staging [13]. As such, pregnant patients with features of ovarian malignancy are a group who require surgical intervention. Alongside increasing the suspicion of malignancy, ovarian masses that are large (>10 cm) or increasing in size pose a risk of torsion or obstructed labour and are an indication for surgery [14]. Another important consideration is the risk of emergency surgery in the event of ovarian torsion and its apparent predisposition towards preterm birth [15].

Such risks must be balanced with the risks inherent to the surgery itself, both to fetus and mother. Delaying surgery until the second trimester affords time for resolution of functional cysts and avoids spontaneous first trimester miscarriage being falsely linked to the operation. Furthermore, organogenesis has largely concluded as well as the pregnancy's reliance upon the corpus luteum for progesterone [1, 16, 17].

In keeping with current practice, we performed the operation in the patient's second trimester for the aforementioned reasons. Additionally, operating before the third trimester reduces the technical burden that a large gravid uterus may place upon the surgeon. Our management included review by our gynaecologic-oncology multidisciplinary team. This is concordant with Vernooij et al. [18] who determined that mean survival time for ovarian malignancy is improved through consultation with a gynaecological oncology service [18].

### 3.2. Laparoscopy and Pregnancy

Multiple studies have corroborated the safety of laparoscopic management of adnexal masses in pregnant patients [19–21]. Compared to an open approach, laparoscopy may mitigate the risks of thromboembolism and maternal hypoventilation by offering earlier mobilisation and less reliance upon postoperative opioid analgesia, respectively [22]. Concerns regarding the risk of preterm birth when operating in the third trimester may be unfounded given that the preterm birth rate ranges between 7.3 and 11.7% in the general population [23, 24].

### 3.3. Pneumoperitoneum

An animal study performed by Barnard et al. [25] demonstrated reduced placental perfusion in the presence of maternal pneumoperitoneum [25]. Encouragingly, fetal perfusion and blood gas values were not adversely affected. These findings are yet to be confirmed or refuted by human studies. In light of its uncertain significance, multiple sources advocate for limiting the operating pneumoperitoneum to below 15 or even 12 mmHg [26–28]. Intra-abdominal pressures should be titrated to account for the already deleterious effect that the gravid uterus has upon visualisation [29].

### 3.4. Electrosurgical Instrumentation

Several case series have incorporated electrosurgical instruments into their laparoscopic surgeries on pregnant women without operative complications [15, 30, 31]. We used the bipolar tissue sealing device Ligasure to excise the cyst, without intraoperative or postoperative complication. The envelopment of the fetus in amniotic fluid is believed to be protective from energy-related injury. As with any application of electrosurgery, care should be taken to avoid inadvertent trauma. This is especially salient in the pregnant patient in whom there is a paucity of evidence regarding its usage [16].

### 3.5. Uterine Manipulation and Tocolysis

Cervical manipulation of the pregnant uterus has the propensity to induce premature contractions, and as such, is contraindicated. Intra-abdominal surgery, particularly in the third trimester, may lead to uterine contractions [32]. However, Walsh et al. [33] demonstrated no reduction in preterm birth with prophylactic tocolysis [33]. As such, we did not manipulate the uterus during the course of our surgery, and tocolysis was not employed.

### 3.6. SILS

SILS is a novel approach to minimal access surgery. A single-entry point is placed, typically at the umbilicus, through which the laparoscope and all instruments are inserted [34]. Its efficacy and safety have been demonstrated across the spectrum of surgical disciplines [35].

Prospective studies comparing SILS to standard laparoscopic management of adnexal lesions are lacking. Several studies have demonstrated the feasibility and safety of SILS for adnexal lesions [36, 37]. A recent retrospective study demonstrated longer operative times when SILS was applied to adnexal lesions [38], whilst shorter operating times have been described by other applications of the technique [39]. Nevertheless, the potential for improved cosmesis and perioperative outcome should not be discounted based on scarcity of evidence.

Delay in widespread adoption of SILS may be in part due to its technical challenges. Convergence of all instruments through one port can reduce the working space available [40]. Furthermore, movement of the camera can be restricted by its proximity to the working instruments [4].

The applicability of SILS to the treatment of a variety of adnexal diseases has been established. Marcelli et al. [41] exhibited good success rates for SILS salpingectomy for ectopic pregnancy. Despite prolonging operative time, patients treated with SILS had shorter hospital stays than those who underwent conventional multiport laparoscopy [41]. Similarly, Loh et al. (2017) found outcomes from SILS management of ectopic pregnancy to be at least equivalent with conventional laparoscopy [42]. Dursun et al. [43] successfully treated 14 women with benign adnexal masses using SILS in combination with standard laparoscopic instruments. Optimisation of instrument and surgeon position compensated for difficulties encountered with instrument collision [43]. In the future, this issue may be avoided entirely with the advent of instruments designed specifically for SILS. Common to existing research surrounding SILS, these studies were marred by small sample sizes. Xiao et al. (2020) have since demonstrated the feasibility of SILS in laparoscopic management of adnexal disease, myomectomy, and cervical cerclage, specifically in the pregnant woman [44].

The importance of cosmesis cannot be understated given that the population who undergo laparoscopy for benign gynaecological disease largely consist of young women. By virtue of a single incision site, SILS has the potential to maximise patient satisfaction postoperatively [45]. Additionally, utilising the umbilicus as the sole incision site limits the potential for immediate and long-term postoperative pain [46].

In our case, due to locomotive restrictions and instrument length size, we were compelled to place an additional port into the left flank to remove the cyst capsule at the end. This was further compounded by the inability to manipulate the uterus due to its gravid state. Our experience is echoed in other reports on the usage of SILS in adnexal cysts. Rezai et al. [47] noted the difficulty faced through not being able to alter the gravid uterus' impact upon the operating field [47]. Also, 24% of patients in a study by Huang et al. [38] required the addition of an accessory port [38]. We were still able to excise the lesion using the SILS port in tandem with the accessory port, avoiding the placement of an additional trocar into the abdomen. It could also be argued that additional port placement after a large cyst decompression in the presence of a gravid uterus gives a safety margin on trauma to the uterus derived from an increased space created as in our case. Bariatric surgery size instruments could be utilised if the concern is instrument size alone. We did not have these available to us.

Ross et al. [48] proposed several potential drawbacks to the use of SILS. The presence of preexisting pelvic adhesions and excessive extra- and intraperitoneal fat may exacerbate the intrinsic difficulties with instrument triangulation [48]. This may render previous surgery and a raised BMI relative contraindications to SILS. However, in our experience, the elevated BMI of the patient did not substantially alter the use of SILS. Moreover, the availability of longer instruments may have averted the need to place an accessory port. This is a technical consideration which could be ameliorated with the development and dispersion of instruments specific to the SILS technique. On the discovery of extensive adhesions or an abdomen that is not amenable to SILS, there is always the recourse to convert to standard laparoscopy.

There is reasonable concern regarding the propensity of SILS to cause umbilical hernia due to the larger incision size for entry. The overall risk of port site hernia is a difficult entity to quantify, a systematic review estimated its prevalence as 0.5% for all laparoscopic surgery [49]. Gunderson et al. (2013) looked specifically at SILS, and adjusting for confounding variables, derived an umbilical hernia rate of 0.5% [50]. Nevertheless, there is not significant literature to reliably refute the hypothetical increased risk of herniation with SILS. In our case, we made a 15 mm incision at the umbilicus. We feel that given that this is only marginally larger than the Hasson entry technique incision the potential benefits of SILS outweigh the theoretical increased risk of umbilical hernia [51]. Overall, the application of SILS reduces the total number of incision sites and potential locations for herniation. Furthermore, fewer trocar insertions expose the patient to less risk of intra-abdominal trauma, bleeding, and pain.

Potential issues with instrument triangulation and obtaining adequate fulcrum in order to manipulate tissue adequately were countered by the flexibility of the Gelseal SILS port. This flexibility affords the surgeon adequate instrument articulation; hence, we do not foresee significant challenges in intracorporeal suturing. Furthermore, the presence of a large volume cyst did not restrict our use of SILS as we decompressed the cyst prior to its removal. We expect that with the increasing prevalence of SILS, adaptations to its unique challenges will proliferate.

## 4. Conclusion

Laparoscopic resection of adnexal lesions is safe during pregnancy and should be favoured over the open approach. SILS minimises incision sites and has potential for reduction in perioperative morbidity. The scope to add additional ports if needed makes SILS a feasible primary approach to laparoscopic cystectomy in the pregnant patient.

## Data Availability

The data presented in this case report is available from the corresponding author upon request.
